# A Double-Edged Sword: The Clinical Dilemma of Lupus Nephritis and Antineutrophil Cytoplasmic Antibody-Associated Vasculitis Overlap Syndrome

**DOI:** 10.7759/cureus.98569

**Published:** 2025-12-06

**Authors:** Letty Probasco, Anja C Fernandez Placencia

**Affiliations:** 1 Rheumatology, Lehigh Valley Health Network, Allentown, USA; 2 Internal Medicine, Ricardo Palma University, Lima, PER

**Keywords:** ana-positive vasculitis, anca-associated vasculitis, crescentic glomerulonephritis, dual glomerulopathy, lupus nephritis, lupus nephritis and aav overlap syndrome, mpo-anca

## Abstract

Lupus nephritis (LN) and antineutrophil cytoplasmic antibody (ANCA)-associated vasculitis (AAV) are classically regarded as distinct autoimmune diseases with differing pathophysiologic mechanisms-immune complex-mediated injury in LN versus pauci-immune necrotizing vasculitis in AAV. However, recent studies have identified a rare but emerging overlap syndrome characterized by dual serologic, histopathologic, and clinical features. This diagnostic entity presents unique challenges, particularly in older adults with rapidly progressive glomerulonephritis (RPGN) and systemic inflammation.

We describe a diagnostically challenging case of a 74-year-old woman with a history of interstitial lung disease, polyarthritis, and sicca symptoms who developed new-onset hemoptysis, peripheral neuropathy, and acute kidney injury. She underwent an extensive workup, which revealed hypocomplementemia, high-titer antinuclear antibody (ANA), and seropositivity for Sjögren's-syndrome-related antigen A (SSA/Ro) and ribonucleoprotein (RNP) antibodies, along with markedly elevated myeloperoxidase-antineutrophil cytoplasmic antibody (MPO-ANCA) levels. These findings raised concern for dual autoimmune pathology. Laboratory evaluation additionally demonstrated worsening kidney function, proteinuria, and microscopic hematuria, prompting a renal biopsy. Histologic examination revealed class IV LN, characterized by diffuse immune complex deposition, as well as necrotizing crescentic glomerulonephritis, a hallmark of pauci-immune AAV. These overlapping serologic and histopathologic features, in the context of pulmonary hemorrhage, were diagnostic of LN-AAV overlap syndrome.

The patient was treated with aggressive immunosuppressive therapy, resulting in improvement of renal function and resolution of systemic symptoms. This case underscores the importance of maintaining a high index of suspicion for overlapping autoimmune phenomena in elderly patients with atypical serologic and histopathologic findings. Early recognition is essential to initiate appropriate therapy and improve outcomes in this complex clinical setting.

## Introduction

Lupus nephritis (LN) is a severe manifestation of systemic lupus erythematosus (SLE) and is characterized by immune complex deposition within the kidneys and complement-mediated glomerular injury [1]. Lupus nephritis is a complication of SLE seen in 30%-40% of cases, often developing early in the disease course and disproportionately affecting young women and children [2]. In contrast, antineutrophil cytoplasmic antibody-associated vasculitis (AAV) is defined as a necrotizing vasculitis with few or no immune deposits that predominantly affects small vessels, associated with myeloperoxidase-directed antineutrophil cytoplasmic antibodies (MPO-ANCA) or proteinase 3-directed antineutrophil cytoplasmic antibodies (proteinase 3-ANCA) [3], with more than 75% of patients having kidney involvement [4]. Although LN and AAV arise from distinct immunopathologic mechanisms, when these two conditions overlap, the underlying immunological environment becomes more aggressive, inducing rapidly progressive organ involvement, particularly of the lungs and kidneys [5]. The earliest biopsy-proven description of this overlap was reported by Marshall et al. in 1997, who documented membranous LN with superimposed pauci-immune necrotizing and crescentic glomerulonephritis [6]. The syndrome was later more fully characterized as a distinct clinicopathologic entity by Nasr et al. in 2008 and has since been described in additional case reports and small series [7].

Patients with SLE-AAV overlap syndrome are mostly female, commonly present with rapidly progressive glomerulonephritis (RPGN), and have both antinuclear antibodies (ANA) and anti-myeloperoxidase (anti-MPO) antibodies. Pulmonary involvement may also occur, including alveolar hemorrhage, which was reported in three of eight patients in one series. Future studies are needed to investigate the possible shared immune pathogenesis that may facilitate the co-occurrence of SLE and AAV in these patients and thus lead to new therapeutic options [8].

Clinicians should consider systemic lupus erythematosus and antineutrophil cytoplasmic antibody disease overlap syndrome when a young female patient presents with new kidney failure and alveolar hemorrhage. Early biopsy and aggressive treatment are essential in preserving kidney function, and plasmapheresis should be considered in severe cases [9]. However, reports in elderly female patients are scarce, making this case relevant as it adds needed clinical detail for this population.

Herein, we describe a diagnostically challenging presentation in a 74-year-old woman with chronic interstitial lung disease (ILD) who developed new pulmonary and renal abnormalities. Her clinical course ultimately reflected overlapping features of LN and AAV, underscoring the need for early recognition and timely immunosuppressive therapy in older adults.

## Case presentation

A 74-year-old Asian woman presented with progressive polyarthritis, peripheral neuropathy, hemoptysis, and dark-colored urine. Her history included gastroesophageal reflux disease and chronic ILD with pulmonary fibrosis, diagnosed in mid-adulthood. Prior records referenced longstanding fibrotic ILD, but the absence of original imaging and serologic data prevented the determination of its etiology.

She also reported a remote diagnosis of rheumatoid arthritis based on earlier serologic testing. Given this autoimmune background, a connective tissue disease-associated interstitial lung disease (CTD-ILD) was clinically suspected as the most plausible etiology of her pulmonary fibrosis; however, this could not be confirmed in the absence of her original diagnostic evaluation. At that time, she had been treated with intermittent courses of corticosteroids for approximately two years, but there was no indication that long-term disease-modifying immunosuppressive therapy had been initiated.

In the weeks preceding her admission, she developed a persistent cough following an upper respiratory infection that failed to improve with antibiotics and a short course of oral corticosteroids. Shortly thereafter, she noted new bilateral knee pain and swelling, progressive tingling in her hands and feet, and marked generalized weakness that limited her mobility. Her son, an internal medicine physician, initiated high-dose prednisone (60 mg daily) for presumed polymyalgia rheumatica or rheumatoid arthritis, which led to rapid symptomatic improvement. However, upon tapering the corticosteroid dose, her joint pain and weakness recurred, and she disclosed new hemoptysis and dark-colored urine, prompting further evaluation.

Review of systems was notable for xerophthalmia and xerostomia, along with morning stiffness involving the small joints of the hands and feet. She denied photosensitivity, malar rash, Raynaud's phenomenon, or pleuritic chest pain. On examination, she was alert and oriented, with no rash, mucosal ulcers, or synovitis. Bibasilar crackles were absent despite her underlying interstitial lung disease. Additional findings included bilateral 2+ lower-extremity edema.

A chest computed tomography (CT) scan obtained during hospitalization demonstrated central perihilar ground-glass opacities, extensive lower-lobe fibrosis, and honeycombing, findings consistent with her previously documented interstitial lung disease, along with a calcified lingular granuloma and a trace posterior pleural effusion. The mediastinum, lymph nodes, and vascular structures appeared normal. The ground-glass opacities were attributed to her recent hemoptysis and suspected diffuse alveolar hemorrhage (DAH) rather than progression of her underlying ILD.

Given the constellation of symptoms and the imaging findings on admission, a comprehensive laboratory evaluation was initiated. Serial complete blood counts (CBCs) demonstrated progressive anemia and thrombocytopenia over the course of hospitalization (Table 1).

**Table 1:** Complete blood count trends Progressive anemia and thrombocytopenia were observed, with nadir hemoglobin and platelet counts recorded on the final day of testing. For each day, two laboratory measurements are presented in chronological order, labeled (1) and (2).

Test	Reference range	Day 1 (1)	Day 1 (2)	Day 2 (1)	Day 2 (2)	Day 3 (1)	Day 3 (2)
Hemoglobin	12.0-16.0 g/dL	7.6	8.3	8.1	8.4	6.6	9.3
Hematocrit	36-46%	22.5	24.5	23.8	24.3	19.1	26.9
White blood cell count (WBC)	4.0-10.0×10³/µL	10.8	11.7	6.5	5.9	6.9	8.6
Red blood cell count (RBC)	3.8-5.2 ×10⁶/µL	2.49	2.67	2.60	2.67	2.10	2.98
Platelet count	150-450 ×10³/µL	103	86	77	80	74	73
Mean platelet volume (MPV)	7.5-11.5 fL	7.8	8.4	8.8	9.2	8.4	8.7
Mean corpuscular volume (MCV)	80-100 fL	90	92	91	91	91	90
Mean corpuscular hemoglobin (MCH)	27-33 pg	30.6	30.9	31.3	31.5	31.3	31.1
Mean corpuscular hemoglobin concentration (MCHC)	32-36 g/dL	33.8	33.7	34.2	34.7	34.4	34.5

Following the hematologic results in Table 1, serum chemistry, renal function, and liver enzyme data are summarized in Table 2. These results demonstrate impaired renal function with reduced estimated glomerular filtration rate (eGFR) and hypoalbuminemia.

**Table 2:** Serum chemistry, renal and liver function Serum creatinine remained above the normal range throughout admission, accompanied by elevated blood urea nitrogen (BUN), hypoalbuminemia, and persistently reduced estimated glomerular filtration rate (eGFR). Mild transaminase elevations were also noted during the hospital course.

Analyte	Reference range	Day 1	Day 2	Day 4	Day 5
Glucose	70-99 mg/dL	92	179	130	101
Blood urea nitrogen (BUN)	7-20 mg/dL	85	89	92	88
Creatinine	0.6-1.3 mg/dL	2.38	2.73	2.68	2.43
Sodium	135-145 mmol/L	135	131	136	135
Potassium	3.5-5.0 mmol/L	4.1	4.7	4.3	4.1
Carbon dioxide (CO₂)	22-29 mmol/L	19	16	17	20
Chloride	98-107 mmol/L	107	105	108	108
Anion gap	8-16	9	10	11	7
Estimated glomerular filtration rate (eGFR)	≥60 mL/min/1.73m²	21	18	18	20
Albumin	3.5-5.0 g/dL	2.5	2.7	2.7	2.5
Calcium	8.5-10.5 mg/dL	8.0	7.8	7.1	6.8
Total protein	6.0-8.3 g/dL	5.1	5.6	5.5	5.2
Total bilirubin	0.1-1.2 mg/dL	1.5	1.2	0.9	0.8
Aspartate aminotransferase (AST)	10-40 U/L	18	21	18	27
Alanine aminotransferase (ALT)	7-56 U/L	11	12	14	25

In conjunction with the renal impairment outlined in Table 2, urinalysis findings (Table 3) demonstrated significant proteinuria, hematuria, and pyuria, with trace bacteriuria interpreted as incidental.

**Table 3:** Urinalysis findings Persistent hematuria, leukocyte esterase positivity, and proteinuria were present, findings consistent with an active glomerular process; trace bacteriuria was noted but not considered clinically contributory. WBC - white blood cell count; RBC - red blood cell count; HPF - high-power field

Analyte	Reference range	Pre-hospitalization 1	Pre-hospitalization 2
Specific gravity	1.005-1.030	1.013	1.020
pH	4.5-8.0	5.0	5.5
Protein	Negative to <30 mg/dL	100-499	100-299
Glucose	Negative	Negative	Negative
Ketone	Negative	Negative	Negative
Blood	Negative	≥1.00	≥1.00
Leukocyte esterase	Negative	500	500
Nitrite	Negative	Negative	Positive
WBC/HPF	0-5 /HPF	>100	>100
RBC/HPF	0-5 /HPF	>100	51-100
Bacteria	None	4+	4+
Mucous threads	None	Few	1+
Squamous epithelial cells	0-5 /HPF	>10	3–5

Urine chemistry studies (Table 4) corroborated the urinalysis findings, demonstrating nephrotic-range proteinuria with a protein-to-creatinine ratio of 3.72 and reduced urinary potassium and chloride excretion.

**Table 4:** Urine chemistry Urine chemistry obtained during hospital admission. Findings demonstrated elevated protein and protein/creatinine ratio, consistent with glomerular disease.

Analyte	Reference range	Day 1
Sodium	40-220 mmol/L	82
Potassium	25-125 mmol/L	20.2
Chloride	110-250 mmol/L	80
Total Protein	Negative to <30 mg/dL	146.2
Protein/creatinine ratio	<0.2 mg/mg	3.72
Creatinine	500-2000 mg/day (spot: variable)	39.3

Given the patient's pulmonary and renal manifestations, an infectious disease workup was performed (Table 5), including viral polymerase chain reaction (PCR) assays, hepatitis and HIV serologies, tuberculosis screening, and blood and urine cultures, all aimed at excluding infectious etiologies.

**Table 5:** Microbiology, viral, hepatitis, and infectious disease testing All viral respiratory polymerase chain reaction (PCR) assays were negative. Serologic testing indicated prior exposure to hepatitis A and hepatitis B, evidenced by reactive hepatitis A total antibody and elevated hepatitis B surface antibody (>500 IU/mL) with negative surface antigen and IgM - consistent with immunity from past infection or vaccination. Blood and urine cultures showed no bacterial growth. EIA - enzyme immunoassay

Test	Reference range / Interpretation	Result
Influenza A, PCR	Not detected	Not detected
Influenza B, PCR	Not detected	Not detected
Respiratory syncytial virus, PCR	Not detected	Not detected
SARS-CoV-2, PCR	Not detected	Not detected
Hepatitis A antibody, IgM	Nonreactive	Nonreactive
Hepatitis A antibody, total	Nonreactive	Reactive
Hepatitis B surface antigen	Nonreactive	Nonreactive
Hepatitis B surface antibody	<10 IU/mL (nonimmune)	>500 IU/mL
Hepatitis B core antibody, total	Nonreactive	Reactive
Hepatitis B core antibody, IgM	Nonreactive	Nonreactive
Hepatitis C antibody (EIA)	Nonreactive	Nonreactive
HIV-1/2 Ag/Ab combo	Nonreactive	Nonreactive
QuantiFERON®-TB Gold Plus	Negative	Negative
Blood cultures	No growth	No growth
Urine cultures	No growth	No growth

With infectious etiologies excluded (Table 5), evaluation shifted to autoimmune serologies. Serologic testing revealed a constellation of markers consistent with systemic autoimmunity and possible ANCA-associated vasculitis overlap (Table 6). The patient demonstrated a high-titer antinuclear antibody (ANA, 1:640, homogeneous pattern) with positive Sjögren syndrome-related antigen A (SSA) and ribonucleoprotein (RNP) antibodies, but negative Sjögren syndrome-related antigen B (SSB) and anti-double-stranded DNA antibody​​​​​​​ (anti-dsDNA) antibodies - a profile often associated with connective tissue disease without classic Sjögren's features. Complement levels were markedly reduced (C3 and C4), and rheumatoid factor was markedly elevated. Notably, myeloperoxidase (MPO)-ANCA was strongly positive, with a p-ANCA titer >1:1280, while proteinase 3 (PR3) antibodies were negative. The extended myositis and systemic sclerosis panels were negative, helping rule out alternative connective tissue disease-associated vasculitides. This antibody profile is atypical but characteristic of LN-AAV overlap syndrome, correlating with renal biopsy findings and systemic manifestations.

**Table 6:** Autoimmune disease panel Comprehensive serologic testing demonstrated depressed complement levels (C3 and C4), elevated rheumatoid factor, and strongly positive MPO-ANCA with high p-ANCA titers. ANA was high-titer and homogeneous, with SSA and RNP positivity. The extended myositis and systemic sclerosis panels were negative. This antibody profile supports overlap between systemic autoimmunity and ANCA-associated vasculitis, consistent with LN–AAV overlap syndrome. Anti-dsDNA - anti-double-stranded DNA antibody; SSA (Ro) - anti-Sjögren syndrome A (Ro) antibody; SSB (La) - anti-Sjögren syndrome B (La) antibody; RNP - ribonucleoprotein; Scl-70 antibody - anti-topoisomerase I antibody; EJ antibody - anti-glycyl-tRNA synthetase antibody; Mi-2 antibody - anti-Mi-2/chromodomain helicase DNA-binding protein antibody; Jo-1 antibody - anti-histidyl-tRNA synthetase antibody; OJ antibody - anti-isoleucyl-tRNA synthetase antibody; PL-7 antibody - anti-threonyl-tRNA synthetase antibody; PL-12 antibody - anti-alanyl-tRNA synthetase antibody; SRP antibody - anti-signal recognition particle antibody; SAE1 antibody - anti-small ubiquitin-like modifier activating enzyme antibody; ANCA IFA - antineutrophil cytoplasmic antibody by indirect immunofluorescence assay; LN -lupus nephritis; ANA - antinuclear antibody; ANCA - antineutrophil cytoplasmic antibody

Test	Reference range	Result
Complement C3	90-180 mg/dL	47
Complement C4	10-40 mg/dL	<8.0
Rheumatoid factor	<30 IU/mL	742
Antinuclear antibody (ANA)	<1:80 (negative)	1:640, homogeneous
Anti-dsDNA antibody	<30 IU/mL	Negative
SSA (Ro) antibody	<20 IU/mL	20
SSB (La) antibody	<20 IU/mL	<20
RNP antibody	Negative	Positive
Sm (Smith) antibody	Negative	Negative
Scl-70 antibody	Negative	Negative
Jo-1 antibody	<20 IU/mL	1
P155/140 antibody	Negative	Negative
EJ antibody	Negative	Negative
Mi-2 antibody	Negative	Negative
OJ antibody	Negative	Negative
PL-7 antibody	Negative	Negative
PL-12 antibody	Negative	Negative
SRP antibody	Negative	Negative
SAE1 antibody	Negative	Negative
Proteinase 3 (PR3) antibody	<20 IU/mL	<4
Myeloperoxidase (MPO) antibody (pre-hospitalization)	<20 IU/mL	179
Myeloperoxidase (MPO) antibody (day 2)	<20 IU/mL	141
Serine protease 3 IgG	<20 IU/mL	3
ANCA IFA titer	<1:20 (negative)	>1:1280
Cyclic citrullinated peptide (CCP) IgG	<20 U/mL	<3.0
Histone antibody	<1.0 U/mL	1.3
RNA polymerase III IgG	<20 U/mL	12
Salivary gland protein 1 antibody, IgG	<20 U/mL	5.9
Salivary gland protein 1 antibody, IgA	<20 U/mL	29.1
Salivary gland protein 1 antibody, IgM	<20 U/mL	14.7

**Figure 1:**
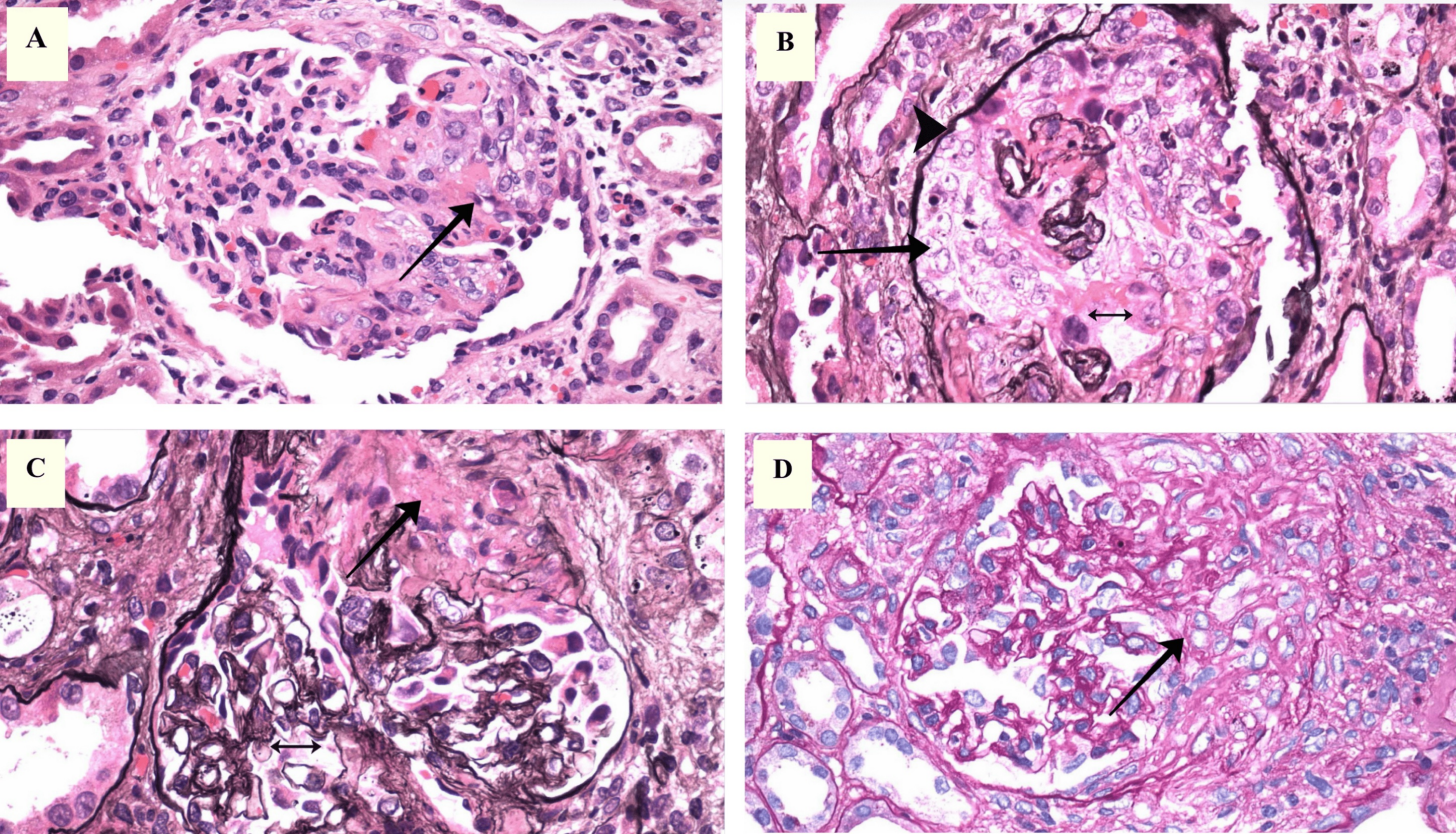
Renal biopsy images on light microscopy in LN-AAV overlap syndrome (A) Hematoxylin and eosin (H&E, ×400): A glomerulus demonstrates marked endocapillary hypercellularity with infiltration by mononuclear inflammatory cells and reactive endothelial proliferation. Segmental fibrinoid necrosis (arrow) of the capillary tuft is present, consistent with pauci-immune glomerulonephritis in antineutrophil cytoplasmic antibody (ANCA)-associated vasculitis. The tubulointerstitium shows inflammatory infiltrates, indicating active immune-mediated injury. (B) Silver stain (×400): A cellular crescent (arrow) partially encircles the glomerular tuft, formed by proliferating parietal epithelial cells. The glomerular basement membrane (GBM; arrowhead) is fragmented (double-headed arrow), confirming active capillaritis. (C) Silver stain (×400): Segmental necrosis (arrow) with obliteration of capillary lumina and architectural disruption. The GBM (double-headed arrow) is ruptured, consistent with pauci-immune crescentic glomerulonephritis. (D) Trichrome stain (×400): Segmental glomerulosclerosis with matrix expansion (arrow). The interstitium shows fibrosis and scattered inflammation, reflecting chronic injury. The combination of necrosis, crescents, and fibrosis supports lupus nephritis–ANCA-associated vasculitis (LN–AAV) overlap syndrome.

**Figure 2:**
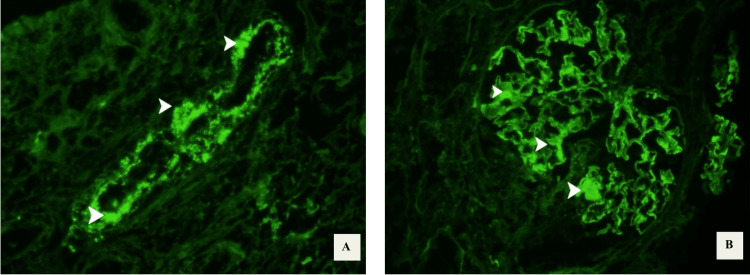
Renal biopsy immunofluorescence findings in LN-AAV overlap syndrome (A) Immunofluorescence demonstrates granular IgG deposition along the glomerular capillary walls (arrowheads), consistent with immune complex–mediated injury. The global granular pattern is characteristic of lupus nephritis. (B) Additional granular immune complex deposition is observed along the tubular basement membranes and interstitial vessels (arrowheads), indicative of tubulointerstitial involvement and vascular immune complex deposition. These findings support active lupus nephritis with concurrent vascular injury in the setting of lupus nephritis–ANCA-associated vasculitis (LN-AAV) overlap.

Given the serologic evidence of systemic autoimmunity and ANCA-positivity (Table 6), along with progressive kidney dysfunction and persistent hematuria, a renal biopsy was pursued early during hospitalization to further characterize the extent of renal involvement. Histopathologic evaluation demonstrated features of both lupus nephritis and ANCA-associated vasculitis. Light microscopy revealed diffuse endocapillary hypercellularity, fibrinoid necrosis, and cellular crescents with disruption of the glomerular basement membrane (Figure 1A-D). Immunofluorescence showed global granular capillary wall staining with immunoglobulin G (IgG) and immune complex deposition along tubular basement membranes and interstitial vessels (Figure 2A-B), confirming a LN-AAV overlap syndrome.

On hospital day four, the renal biopsy was performed after three days of pulse intravenous methylprednisolone and transition to oral prednisone at 1 mg/kg. A perinephric hematoma was noted on ultrasound performed afterward, a finding commonly observed following percutaneous renal biopsy. Around the same time, the patient's hemoglobin decreased from 8.9 g/dL to 7.1 g/dL and then to 6.7 g/dL, while platelet counts remained stable at approximately 72,000/µL. She received one unit of packed red blood cells, increasing hemoglobin to 9.5 g/dL.

Laboratory evaluation demonstrated findings consistent with microangiopathic hemolytic anemia (MAHA), including elevated lactate dehydrogenase (347 U/L), undetectable haptoglobin (<3 mg/dL), the presence of schistocytes on peripheral smear, and reduced ADAMTS13 activity (35.1%).

On hospital day seven, therapeutic plasma exchange was initiated and administered every other day for a total of five sessions. Immunosuppression was escalated with cyclophosphamide at 15 mg/kg initiated shortly after the start of plasma exchange, with subsequent doses administered at standard clinical intervals. Renal function improved thereafter, stabilizing at a new baseline creatinine of 1.4 to 1.5 mg/dL.

After hospital day seven, hemoptysis developed at a time when corticosteroids, plasma exchange, and cyclophosphamide were already in progress. Chest CT demonstrated central perihilar ground-glass opacities superimposed on chronic fibrotic changes, findings concerning for DAH associated with ANCA-associated vasculitis and LN. Because she was already receiving high-dose corticosteroids, cyclophosphamide, and plasma exchange, bronchoscopy was deferred. Her respiratory symptoms improved with the continuation of therapy.

She was started on atovaquone for Pneumocystis jirovecii pneumonia (PJP) prophylaxis and pantoprazole for gastrointestinal protection. Infectious disease evaluation identified isolated hepatitis B core antibody positivity with negative surface antigen, and antiviral prophylaxis was not recommended. The treatment plan included ongoing cyclophosphamide every three weeks for up to six months, tailored to clinical and serologic response.

## Discussion

Lupus nephritis and antineutrophil cytoplasmic antibody-associated vasculitis are regarded as distinct autoimmune processes, with LN driven by immune complex-mediated glomerular injury [1] and AAV characterized by pauci-immune necrotizing vasculitis [3]. Nonetheless, a growing body of literature describes patients who exhibit overlapping serologic, histopathologic, and clinical features, leading to increased recognition of LN-AAV overlap syndrome as a rare but important diagnostic entity [4-8].

Our patient demonstrated multiple serologic markers of systemic autoimmunity, including a high-titer ANA (1:640, homogeneous pattern), SSA and RNP antibodies, and marked hypocomplementemia. Her presentation was further complicated by a strongly positive MPO-ANCA with a p-ANCA titer >1:1280, while PR3 antibodies remained negative - a pattern that, although atypical, is increasingly recognized in LN-AAV overlap. Mixed autoimmune serologies such as these have been described across multiple reports of overlap syndromes and raise suspicion for dual pathology when accompanied by renal dysfunction [9-11].

The renal biopsy provided definitive confirmation of overlap pathology. Diffuse endocapillary hypercellularity and granular immune complex deposition were characteristic of LN, while fibrinoid necrosis and cellular crescents were consistent with necrotizing AAV. The coexistence of immune complex-mediated and pauci-immune lesions, as seen in this case, has been increasingly reported in LN-AAV overlap syndromes and may be associated with more aggressive renal injury [4-9,12,13]. Imaging further supported systemic involvement: although prior films were unavailable, chest CT on admission demonstrated chronic interstitial changes along with new ground-glass opacities attributed to diffuse alveolar hemorrhage, a complication well described in overlap scenarios [5,8,12,14].

Older adults presenting with LN-AAV overlap may show atypical or muted manifestations, as highlighted in a 77-year-old reported case [12]. Some presentations may be clinically silent, contributing to diagnostic delay and obscuring disease evolution [9]. Multiple studies emphasize that serologic and histopathologic findings may evolve or diverge over time, complicating early diagnosis [9-11]. LN-AAV overlap has also been consistently associated with severe renal manifestations, including necrotizing and crescentic lesions and rapid deterioration in kidney function [6-12,14]. Pulmonary involvement, ranging from interstitial lung disease to diffuse alveolar hemorrhage, is likewise documented across overlap cases and reflects the extent of systemic capillaritis [5,8,12,14]. Our patient's course was further complicated by a perinephric hematoma and microangiopathic hemolytic anemia (MAHA), findings previously reported in severe presentations and indicative of extensive endothelial injury [5].

Management of LN-AAV overlap is not standardized due to its rarity. Most published cases describe induction regimens extrapolated from treatment approaches for severe LN and AAV, typically combining high-dose glucocorticoids with cyclophosphamide or rituximab [4,14]. In this case, induction with corticosteroids, cyclophosphamide, and plasmapheresis resulted in stabilization of renal function and improvement in systemic features. Although the benefit of plasmapheresis remains debated, it is often considered in settings of diffuse alveolar hemorrhage, severe renal injury, or MAHA [4,14]. Its potential benefit may derive from rapid removal of circulating MPO-ANCA, immune complexes, and pro-inflammatory mediators, thereby reducing ongoing endothelial injury in severe vasculitis presentations [4,9,13,14]. Interpretation of her chronic ILD remained limited by the absence of prior imaging or baseline pulmonary studies; however, ILD has been increasingly described in AAV and in LN-AAV overlap presentations [5,12,14].

Timely recognition of LN-AAV overlap is essential, particularly in older adults with pulmonary-renal manifestations or discordant autoimmune serologies. The combination of hypocomplementemia, MPO-ANCA positivity, and mixed glomerular histopathology underscores the central role of kidney biopsy in establishing the diagnosis. Prompt initiation of aggressive immunosuppression remains critical to preventing irreversible organ damage. In acute presentations, treatment should prioritize control of severe organ-threatening disease without delaying therapy to differentiate LN, AAV, or overlap; however, establishing the overlap diagnosis becomes crucial once the patient is stabilized, as it guides long-term immunosuppression, monitoring, and prognosis. Incorporation of a multidisciplinary approach, including early nephrology and rheumatology consultation, may facilitate earlier recognition in patients whose serologic or clinical features do not align with a single autoimmune process.

This case also illustrates several limitations. The absence of prior laboratory data and historical lung imaging restricted assessment of her autoimmune trajectory and the chronicity of her interstitial lung disease. Serologic evolution before hospitalization could not be evaluated, and therapeutic decisions, such as initiating plasmapheresis, were based on clinical severity rather than standardized guidelines. These limitations reflect real-world challenges in diagnosing and managing LN-AAV overlap and highlight the need for greater awareness of this entity across primary and specialty care settings.

## Conclusions

Identifying the underlying cause of renal dysfunction in patients with complex autoimmune presentations requires careful integration of clinical, serologic, imaging, and histopathologic data. LN-AAV overlap remains a rare but important diagnostic consideration, particularly in older adults with pulmonary-renal manifestations or discordant autoimmune markers. In this case, the combination of immune complex-mediated lupus nephritis and pauci-immune necrotizing glomerulonephritis was confirmed by kidney biopsy and supported by MPO-ANCA positivity and hypocomplementemia. Timely initiation of appropriate immunosuppressive therapy, including high-dose corticosteroids, cyclophosphamide, and adjunctive plasmapheresis, resulted in stabilization of renal function and improvement in systemic manifestations. Early biopsy and aggressive treatment remain essential to preventing irreversible organ damage in patients with suspected LN-AAV overlap.
